# Is Fecal Calprotectin an Applicable Biomarker of Gut Immune System Activation in Chronic Inflammatory Demyelinating Polyneuropathy? – A Pilot Study

**DOI:** 10.3389/fnhum.2021.733070

**Published:** 2021-11-12

**Authors:** Magdalena Koszewicz, Agata Mulak, Edyta Dziadkowiak, Sławomir Budrewicz

**Affiliations:** ^1^Department of Neurology, Wrocław Medical University, Wrocław, Poland; ^2^Department of Gastroenterology and Hepatology, Wrocław Medical University, Wrocław, Poland

**Keywords:** chronic inflammatory demyelinating polyneuropathy, intestinal inflammation, fecal calprotectin, gut immune system, gut microbiota

## Abstract

**Introduction:** Chronic inflammatory demyelinating polyneuropathy (CIDP) is a complex autoimmune disease caused by dysregulated response to not fully recognized antigens. Some association between CIDP and inflammatory bowel disease (IBD) has been reported, but the exact pathophysiological links of these disorders are not well understood.

**Aim of the Study:** To evaluate fecal calprotectin as a biomarker of gut inflammation in CIDP patients without IBD.

**Methods:** Fifteen patients with CIDP and 15 healthy controls were included in the study. The CIDP diagnosis was based on the EFNS/PNS criteria. The occurrence of bowel symptoms was assessed based on a questionnaire. The quantitative evaluation of fecal calprotectin level was performed by the ELISA test.

**Results:** The fecal calprotectin level (μg/g) expressed as median along with the lower and upper quartiles [25Q–75Q] was significantly higher in CIDP patients compared to the controls: 26.6 [17.5–109.0] vs 15.6 [7.1–24.1], *p* = 0.0066. Abnormal fecal calprotectin level (>50 μg/g) was found in 33% of all CIDP patients and in none of the control subjects. The patients with abnormal fecal calprotectin level did not differ from the rest of the study group regarding the neurological status. The most common bowel symptoms reported by CIDP patients included constipation (33%), feeling of incomplete evacuation (33%), bloating (27%), and alternating bowel movement pattern (27%).

**Conclusion:** In one-third of CIDP patients the signs of gut immune system activation have been observed. This finding may be associated with CIDP pathogenesis and induction of autoimmune response as well as concomitant dysautonomia with gastrointestinal symptoms.

## Introduction

Chronic inflammatory demyelinating polyneuropathy (CIDP) is characterized by symmetrical, proximal and/or distal paresis as well as sensory loss developing over a period of at least 8 weeks (Viala et al., 2010). CIDP is a complex autoimmune disease with two or more distinctive pathophysiological mechanisms (Dimachkie and Barohn, 2013; Roggenbuck et al., 2018). Although macrophage-induced demyelination is the pathological characteristic in the majority of CIDP patients, recently, nodal and paranodal IgG4 autoantibodies against different antigens including neurofascin-186, neurofascin-155, contactin-1, and contactin-associated protein 1 have been described in classic CIDP as well as in some of its atypical forms (Lehmann et al., 2019; Koike and Katsuno, 2020; Ogata, 2020).

Currently, there is a growing recognition of the role of gut inflammation, gut dysbiosis and altered intestinal permeability in the pathogenesis of different autoimmune diseases (Campbell, 2014). Since the gut microbiota is a key regulator of immune responses, in many chronic inflammatory diseases the gut microbial imbalance might be a trigger (Blaser and Falkow, 2009; Belkaid and Hand, 2014). Some association between CIDP and inflammatory bowel disease (IBD) including ulcerative colitis and Crohn’s disease (CD) has been reported, but the exact pathophysiological links of these entities are not well understood (Ariatti et al., 2009). Although antigen specificity remains unknown, a concurrent interaction of T cell-mediated and humoral autoimmunity might be considered in both disorders (Ohyagi et al., 2013). It has been suggested that the autoimmune attack against distinct components of peripheral nerves and gut tissue might be triggered by some microbial antigens due to molecular mimicry (Kovvali and Das, 2005; Yuki, 2007). Additionally, gut dysbiosis may induce gut inflammation and increased intestinal barrier permeability contributing to the development of autoimmune response not only at the gut level but also other organs and systems (Campbell, 2014). Many extraintestinal symptoms observed in IBD confirm such an association. Noteworthy, peripheral neuropathy is one of the most frequently reported neurologic complications of IBD (Gondim et al., 2005). The observed frequency of polyneuropathy in IBD patients varies significantly depending on the study population characteristics as well as neuropathy criteria and may reach up to 39% (Sassi et al., 2009; Shen et al., 2012; Figueroa et al., 2013). CIDP-like neuropathy can be an initial presentation of CD, whereas successful treatment of CD may lead to recovery from CIDP (Kim et al., 2015).

Fecal calprotectin, the main protein produced by activated monocytes and neutrophils in the inflamed gut tissue, serves as a key marker for the level of intestinal inflammation (Stríz and Trebichavský, 2004). It is routinely used in diagnosis and monitoring of IBD. Recognizing the previously reported link between CIDP in IBD, this study was aimed to verify if signs of gut immune system activation assessed by fecal calprotectin level are also present in patients with CIDP without concomitant IBD. Additionally, the occurrence of bowel symptoms in the course of CIDP was assessed.

## Materials and Methods

The study was conducted in 15 patients with CIDP (mean age 64, range 52–70 years) hospitalized at the Department of Neurology at Wrocław Medical University (Poland) and 15 healthy controls (mean age 59, range 46–75 years). The CIDP diagnosis was based on the EFNS/PNS criteria (Joint Task Force of the EFNS and the PNS, 2010). Data on comorbidities, pharmacotherapy and gastrointestinal symptoms were assessed based on a questionnaire. The predominant stool type and mean level of abdominal pain intensity were evaluated using the Bristol Stool Form Scale and the Visual Analog Scale (VAS), respectively. The exclusion criteria included IBD and symptomatic diverticulosis, previous gastrointestinal surgery except for appendectomy and cholecystectomy, and use of non-steroidal anti-inflammatory drugs or antibiotics within the last month prior the stool collection. Patients with other polyneuropathies, diseases and conditions influencing the peripheral nervous system, i.e., malignancy, vitamin deficiency, exposure to toxins, drug and alcohol addiction were also excluded. Concomitant autoimmune disorders did not constitute an exclusion criterium. All patients had typical CIDP. None of them were treated with intravenous immunoglobulins previously, but 3 of them were given corticosteroids in the past. Demographic and clinical characteristics of CIDP patients are presented in Table 1. From each subject participating in the study a stool sample was obtained. All samples were delivered to the laboratory within 24 h and after required preparation stored at −20°C until processing as described previously (Ohlsson et al., 2017). The quantitative evaluation of calprotectin in stool samples was performed by ELISA test EK-CAL (Bühlmann Laboratories, Switzerland).

**TABLE 1:** Demographic and clinical characteristics of the recruited CIDP patients.

	CIDP patients *n* = 15	
	Normal fecal calprotectin (<50 μg/g) *n* = 10	Increased fecal calprotectin (≥50 μg/g) *n* = 5
Mean age (years)	64	62
M/F	9/1	4/1
CIDP duration (range, years)	2–12	1–11
EFNS/PNS criteria definite/probable	4/1	7/3
Protein level in CSF (range, mg/dl)	50–231	40–121
Other autoimmune disease	1	1
Abnormal bowel habit (yes/no)	5/5	4/1
Feeling of incomplete evacuation (yes/no)	3/7	2/3
Bloating (yes/no)	3/7	1/4

*CSF, cerebrospinal fluid; EFNS/PNS, European Federation of Neurological Societies/Peripheral Nerve Society; F, female; M, male.*

The protocol of this study was approved by the local Ethics Committee (KB-491/2017). A written informed consent in accordance with the Declaration of Helsinki was obtained from all participants prior to the study enrollment.

Non-parametric statistics were used and results are expressed as median along with the lower and upper quartiles [25Q–75Q]. The Mann-Whitney *U* test was applied to compare differences in fecal calprotectin levels between the groups.

## Results

**FIGURE 1:**
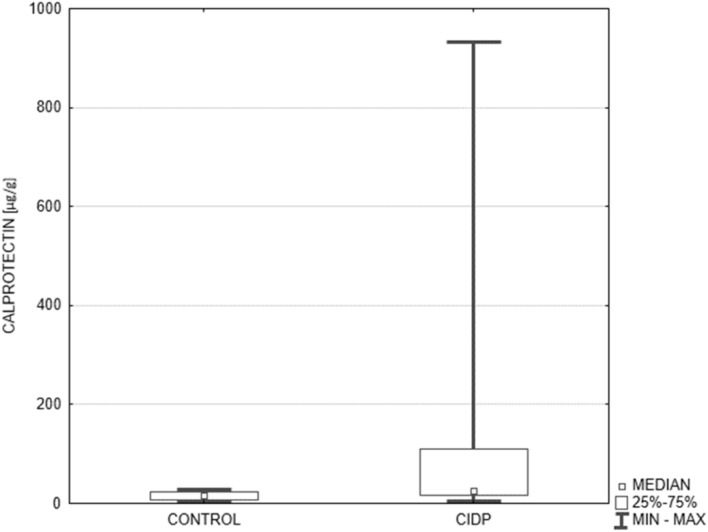
Comparison of fecal calprotectin level in the control group and CIDP patients. Median fecal calprotectin level (μg/g) expressed as median along with the lower and upper quartiles was significantly higher in CIDP patients (*n* = 15) compared to the controls (*n* = 15): 26.6 [17.5–109.0] vs 15.6 [7.1–24.1], *p* = 0.0066. CIDP, chronic inflammatory demyelinating polyneuropathy (Mann-Whitney *U* test).

The diagnostic criteria for definite and probable CIDP were fulfilled in 11 and 4 patients, respectively. The mean duration of CIDP was 4.5 years (range 1–12 years). The mean protein level in cerebrospinal fluid (CSF) amounted to 94 mg/dl (range 40–231 mg/dl). The fecal calprotectin level (μg/g) was significantly higher in CIDP patients compared to the controls: 26.6 [17.5–109.0] vs 15.6 [7.1–24.1], *p* = 0.0066 (Figure 1). Applying the cut-off value of 50 μg/g, abnormal fecal calprotectin level was found in 33% of all CIDP patients and in none of the control subjects.

The most common bowel symptoms reported by the CIDP patients included abnormal bowel habit (67%), feeling of incomplete evacuation (33%), bloating (27%), and abdominal pain (7%). Normal bowel movement pattern was observed only in 33% of the studied CIDP patients. Two-third of them experienced changes in stool consistency and frequency such as constipation (33%), alternating bowel movement pattern (27%), and diarrhea (7%).

Comparing basic data of the patients with normal and increased fecal calprotectin level presented in Table 1 no evident differences were observed with respect to neurological symptomatology, CIDP classification and duration, as well as CSF protein level. Only in two CIDP patients concomitant autoimmune diseases were present. One of the female patients with Hashimoto’s disease had elevated fecal calprotectin level (109 μg/g), whereas in one male patient with systemic lupus erythematosus calprotectin level was within the normal range (42.3 μg/g).

## Discussion

The results of the present study indicate that one-third of CIDP patients without any recognized bowel disease are characterized by the signs of gut immune system activation reflected by the increased fecal calprotectin level. Although the median fecal calprotectin level in all studied CIDP patients was still within the normal range (i.e., below 50 μg/g) it was significantly higher compared to that in the controls (26.6 vs 15.6 μg/g, *p* = 0.0066).

Fecal calprotectin being a complex of two calcium binding proteins S100A8 and S100A9 constitutes up to 60% of the soluble protein content of the neutrophil cytosol and exerts bacteriostatic and fungistatic properties (Walsham and Sherwood, 2016). Intestinal inflammation is associated with migration of neutrophils to the intestinal mucosa or even to the gut lumen in case of gut barrier disturbances. The main function of S100A8 and S100A9 proteins is the regulation of leukocyte chemotaxis and tissue infiltration (Sunahori et al., 2006). Therefore, fecal calprotectin serves as a marker of intestinal inflammation commonly used in clinical practice mainly to diagnose and monitor IBD but also to detect signs of the gut immune system activation in other diseases including autoimmune and neurological disorders. For example, increased fecal calprotectin level has been reported in systemic sclerosis (Marie et al., 2015) and ankylosing spondylitis (Klingberg et al., 2019), Parkinson’s disease (Schwiertz et al., 2018; Mulak et al., 2019), Alzheimer’s disease (Leblhuber et al., 2015), or multiple sclerosis (Buscarinu et al., 2018). Noteworthy, the S100A8 and S100A9 proteins have intrinsically amyloidogenic amino acid sequences and can form amyloid oligomers and fibrils resembling amyloid polypeptides such as α-synuclein and amyloid β (Kowalski and Mulak, 2019). Calprotectin acts not only as an endogenous activator of innate immune response, but may also exert a regulatory function in the adaptive immune system (Ometto et al., 2017). It is involved in the induction of autoreactive CD8 + T cells (Loser et al., 2010). In a murine model of autoimmunity, the lack of S100A8 and S100A9 was associated with a reduction of IL-17 release from autoreactive CD8 + T cells and with lower autoantibody production (Loser et al., 2010). Moreover, it has been shown that calprotectin is the endogenous ligand of CD69 expressed on regulatory T cells participating in the CD4 + T cell differentiation into regulatory T cells (Chih-Ru et al., 2015). Additionally, calprotectin regulates cytokine production including anti-inflammatory transforming growth factor ß (Chih-Ru et al., 2015).

One of the key regulators of immune homeostasis is the gut microbiota (Hemarajata and Versalovic, 2013). Gut dysbiosis is directly associated with the gut immune system activation and disturbances in the intestinal barrier permeability (Campbell, 2014). Changes in the intestinal permeability may promote translocation of bacteria and endotoxins across the epithelial barrier. It induces immunological response associated with the production of pro-inflammatory cytokines resulting in systemic inflammation and subsequent neuroinflammation (Campbell, 2014).

Macrophage-induced demyelination is regarded to be the most important mechanism in the pathogenesis of CIDP. It was found both in typical and atypical forms of CIDP. The trigger factors for the myelin phagocytosis remain unknown. The second possible mechanism is nodo-paranodopathy. IgG4 antibodies against protein at the nodes of Ranvier and paranodal region were detected in typical, as well as atypical CIDP subtypes (mainly distal acquired demyelinating symmetric polyneuropathy – DADS). In typical CIDP proximal and distal nerve segments are involved, and humoral immunity seems to dominate, but patients with nodo-paranodopathy have more significant electrophysiological changes, and they are resistant to intravenous immunoglobulin treatment (Lehmann et al., 2019; Koike and Katsuno, 2020; Ogata, 2020).

While the origin of CIDP still remains not fully recognized, there is a growing understanding of the role of gut dysbiosis together with genetic predisposition and environmental factors (e.g., infections) in the development of autoimmune diseases (Rook and Brunet, 2005). The suggested role of gut inflammation, at least in a subset of CIDP patients, may have important clinical implications. In the presented research the activation of gut immune system was confirmed in about 30% patients with CIDP. All patients had the typical form of the disease. Regarding the CIDP duration, clinical classification, and CSF protein level, they did not differ from the group with normal calprotectin level. However, due the small number of subjects the clear relationship between calprotectin level and clinical presentation, and severity of the disease cannot be established.

The modulation of the gut microbiota composition has been found to exert therapeutic effect in many autoimmune disorders including IBD (Sheil et al., 2007), type 1 diabetes (Calcinaro et al., 2005), systemic lupus erythematosus (Alard et al., 2009), multiple sclerosis (Lavasani et al., 2010), or Parkinson’s disease (Gazerani, 2019). Noteworthy, probiotics have been also suggested as a potential therapeutic option in Guillain-Barré syndrome (Saxena, 2016). The role of probiotics in CIDP has not been investigated so far. Empirical evidence on the role of gut microbiota and gut immune system activation at least in a subset of CIDP is provided by few case reports on peripheral neuropathy after fecal microbiota transplantation (FMT) that have previously been reported (Didesch et al., 2016).

The majority of CIDP patients in the current study suffered from abnormal bowel habit, bloating, and some anorectal sensory disturbances manifesting as feeling of incomplete evacuation. The reported gastrointestinal symptoms might be associated with the gut immune system activation potentially related to concomitant gut dysbiosis. However, these symptoms might also result from autonomic dysfunction which is common in CIDP (Ishii et al., 2005; Figueroa et al., 2012). The prevalence of autonomic dysfunction in CIDP ranges from 21 to 86%, but it is mostly transient and rather mild or even subclinical (Pasangulapati et al., 2017). Interestingly, a case of incomplete intestinal obstruction as an initial and persistent presentation in CIDP has been recently reported (Wang et al., 2018). It has been suggested that circulating antibodies may damage sympathetic and parasympathetic neurons (Querol et al., 2017). In Guillain-Barré syndrome antibodies targeting monoaminergic neurons have been described independently (Lehmann et al., 2010; Rink et al., 2017).

The small number of subjects included in this pilot study is one of the main limitations of the analysis. Nevertheless, the obtained results highlight a novel research field associated with close interactions between the gut immune system and inflammatory peripheral neuropathies. Moreover, since there is evidence that a number of immunomodulators, including corticosteroids, may reduce calprotectin expression (Ometto et al., 2017), it would be crucial to evaluate changes in fecal calprotectin level after immunomodulatory treatment in CIDP patients in a prospective study. Calprotectin can be suggested as a candidate biomarker for the follow-up of disease activity in many autoimmune disorders, since it can predict response to treatment or disease relapse (Pruenster et al., 2016).

In conclusion, the results of the current study confirmed signs of the gut immune system activation in a significant part (30%) of CIDP patients that may be associated with the pathogenesis of the disease and induction of autoimmune response as well as with gastrointestinal symptoms frequently observed in CIDP patients. Further studies aimed at elucidating the link of gut inflammation in CIDP may support immunomodulatory therapeutic approach including the gut microbiota modification.

## Data Availability Statement

The raw data supporting the conclusions of this article will be made available by the authors, without undue reservation.

## Ethics Statement

The studies involving human participants were reviewed and approved by the Ethics Committee at Wrocław Medical University, Wrocław, Poland (KB-491/2017). The patients/participants provided their written informed consent to participate in this study.

## Author Contributions

MK and AM designed the study and wrote the manuscript. ED collected the samples. SB was involved in writing and revising the manuscript. All authors were involved in collecting and interpreting the data and approved the final version of the article.

## Conflict of Interest

The authors declare that the research was conducted in the absence of any commercial or financial relationships that could be construed as a potential conflict of interest.

## Publisher’s Note

All claims expressed in this article are solely those of the authors and do not necessarily represent those of their affiliated organizations, or those of the publisher, the editors and the reviewers. Any product that may be evaluated in this article, or claim that may be made by its manufacturer, is not guaranteed or endorsed by the publisher.
